# Innovations in Dementia Empowerment and Action [IDEA]: First Dementia/Memory Loss RCT Intervention for Sexual and Gender Minority Older Adults and Their Care Partners

**DOI:** 10.1002/alz.091558

**Published:** 2025-01-09

**Authors:** Karen I Fredriksen‐Goldsen, Hyun‐Jun Kim, Brittany R Jones, Charles Pitre Hoy‐Ellis, Austin G. Oswald, Linda Teri

**Affiliations:** ^1^ University of Washington, Seattle, WA USA; ^2^ Northwest Research Group on Aging, Seattle, WA USA

## Abstract

**Background:**

Sexual and gender minority (SGM) older adults experience disparities in subjective cognitive impairment when compared to heterosexuals of similar age. Yet, SGM older adults and their care partners living with dementia/memory loss face substantial barriers to accessing culturally relevant health and dementia care. To address the gap in culturally relevant interventions, we tested the efficacy of a first of its kind stigma reduction, cognitive behavioral intervention for SGM older adults living with dementia/memory loss and their care partners, Aging with Pride: Innovations in Empowerment and Action (IDEA). IDEA is a 6‐week, 9‐session culturally responsive intervention delivered by trained coaches built upon an efficacious dementia intervention, Reducing Disability in Alzheimer’s Disease (RDAD).

**Methods:**

Using a 2‐arm, single‐blind randomized controlled trial with a staggered multiple baseline design, we enrolled 161 dyads of SGM older adults living with dementia/memory loss and their care partners and randomized them to either the IDEA or RDAD intervention arms. For this presentation, we tested the short and long‐term effects in exercise, physical functioning, and depression, as well as the moderating effects of social support and microaggressions. We used linear mixed models with random intercepts and slopes to analyze our longitudinal data.

**Results:**

Among the IDEA participants, those living with dementia/memory loss showed a significant increase in the number of days exercising post‐intervention, and care partners had decreased depression. The treatment effects for exercise among care recipients persisted at 30 weeks post‐intervention, while the treatment effects for depression among care partners persisted at both 30‐ and 52‐weeks post‐intervention. We also found moderating effects of social support and microaggressions on key outcomes.

**Conclusions:**

IDEA, designed to be culturally tailored for underserved and stigmatized populations, including SGM older adults and care partners, was found to be an efficacious intervention. The IDEA intervention is poised to be responsive to the disparities in dementia care in our increasingly diverse and growing older adult population. Recognizing the importance of cultural responsiveness and stigma reduction in dementia and caregiving interventions, the study findings will be disseminated among care providers and within underserved communities.

